# Microstructure Evolution and Mechanical Properties of Melt Spun Skutterudite-based Thermoelectric Materials

**DOI:** 10.3390/ma13040984

**Published:** 2020-02-22

**Authors:** Huiyuan Geng, Jialun Zhang, Tianhong He, Lixia Zhang, Jicai Feng

**Affiliations:** State Key Laboratory of Advanced Welding and Joining, Harbin Institute of Technology, Harbin 150001, China; genghuiyuan@hit.edu.cn (H.G.); jialunzhang@sohu.com (J.Z.); TianhonHe@sohu.com (T.H.); JicaiFeng@sohu.com (J.F.)

**Keywords:** skutterudite alloys, rapid solidification, thermoelectric performance, mechanical strength

## Abstract

The rapid solidification of melt spinning has been widely used in the fabrication of high-performance skutterudite thermoelectric materials. However, the microstructure formation mechanism of the spun ribbon and its effects on the mechanical properties are still unclear. Here, we report the microstructure evolution and mechanical properties of La–Fe–Co–Sb skutterudite alloys fabricated by both long-term annealing and melt-spinning, followed by sintering approaches. It was found that the skutterudite phase nucleated directly from the under-cooled melt and grew into submicron dendrites during the melt-spinning process. Upon heating, the spun ribbons started to form nanoscale La-rich and La-poor skutterudite phases through spinodal decomposition at temperatures as low as 473 K. The coexistence of the micron-scale grain size, the submicron-scale dendrite segregation and the nanoscale spinodal decomposition leads to high thermoelectric performance and mechanical strength. The maximum three-point bending strength of the melt spinning sample was about 195 MPa, which was 70% higher than that of the annealed sample.

## 1. Introduction

Sb-based filled skutterudite [1,2,3] alloys (RTM_4_Sb_12_, R = rare earth element, TM = Co, Fe, etc.) are among the most promising medium temperature thermoelectric materials [4,5,6,7,8], which can directly convert heat to electricity, and vice versa. The efficiency of the thermoelectric material is governed by the dimensionless figure of merit *ZT* = S^2^σT/κ, where S, σ, T and κ are the Seebeck coefficient, electrical conductivity, absolute temperature and thermal conductivity, respectively. Filling the structural void of skutterudite alloys with rare earth elements, such as La, Ce and Yb etc., can greatly suppress the lattice thermal conductivity while keeping a relatively high power factor (PF = S^2^σ). Meanwhile, the filled skutterudites possess relatively high mechanical strength and thermal stability.

Despite the advantages of these physical and mechanical properties, it is quite difficult to obtain high phase purity skutterudite alloys using the traditional solidification approaches [9,10,11,12,13,14]. The reason is that the solidification of skutterudite alloys undergoes two peritectic reactions. Instead, the melt-spinning [15,16,17] followed by hot-pressing approaches are usually used to synthesize bulk skutterudite alloys; high *ZT*s are obtained in these bulk samples [18,19,20,21,22,23,24,25]. For instance, previous studies [26,27,28] reveal that a high-density coherent interface strain field can be generated by the spinodal decomposition in the *p*-type skutterudite, La_0.8_Ti_0.1_Ga_0.1_Fe_x_Co_4–x_Sb_12_, fabricated by the melt-spinning combined with the hot-pressing approaches. The coherent interface strain field causes a conduction band edge fluctuation, multiscale strain field fluctuation and formation of nanostructures, leading to a reduced bipolar effect and wide-frequency spectrum phonon scattering. These features result in the highest conversion efficiency in *p*-type lead-free thermoelectric materials. However, most of the research has focused on the thermoelectric properties of the fabricated samples. The effects of rapid solidification on the microstructure of the skutterudite samples, as well as their mechanical properties, are still unclear. The thermoelectric properties, in turn, are linked to the size and distribution of the strain field generated by the spinodal decomposition in the melt-spinning samples. Therefore, better understanding and control of the rapid solidification and hot-pressing processes must be obtained to further improve the thermoelectric and mechanical performance of the filled skutterudites.

In this paper, the mechanism of the peritectic transition and the spinodal decomposition in the La_0.8_Ti_0.1_Ga_0.1_Fe_3_CoSb_12_ system and its effects on the three-point bending strength have been studied. Temperature-dependent X-ray diffraction and high-resolution transmission electron microscopy (HRTEM) revealed that rapid solidification played a vital role in the formation of the final microstructure of the sintered samples. The peritectic skutterudite phase nucleated directly from the under-cooled melt and grew into submicron dendrites during the melt-spinning process; the coexistence of the submicron-scale dendrite segregation and nanoscale spinodal decomposition led to multiscale strain field fluctuation in the hot-pressed sample, which possessed high thermoelectric performance. The maximum three-point bending strength of the melt-spinning sample was found to be 70% higher than that of the annealed one.

## 2. Materials and Methods 

La ingot (99.8%, Alfa Aesar, Ward Hill, MA, USA), Ti ingot (99.995%, Alfa Aesar, Ward Hill, MA, USA), Ga ingot (99.999%, Alfa Aesar, Ward Hill, MA, USA), Sb balls (99.999%, Alfa Aesar, Ward Hill, MA, USA), Co ingot (99.95%, Alfa Aesar, Ward Hill, MA, USA) and Fe ingot (99.99%, Alfa Aesar, Ward Hill, MA, USA) were weighted according to the chemical composition of La_0.8_Ti_0.1_Ga_0.1_Fe_3_CoSb_12_ and loaded into a carbon crucible, and then were sealed in quartz tubes under vacuum below 10^−3^ Pa. Here, Ti and Ga were introduced to enhance the total filling fraction of the fillers and to stabilize the *p*-type skutterudite crystal [29,30]. The quartz tubes were heated to 1373 K at a speed of 10 K min^−1^ and kept for 4 h to obtain the smelted ingot. One smelted ingot was annealed at 973 K for 100 h and ground into fine powders (<50 µm) and then sintered using the DC hot-pressing at 923 K for 5 min under a pressure of 60 MPa, and named annealed samples. Another smelted ingot with the same chemical composition was put into a quartz tube with a 0.5 mm diameter nozzle, melted by high-frequency induction, and injected under a pressure of 0.05 MPa high purity Ar onto a copper wheel rotating with a linear speed of 60 m s^−1^. The melt-spinning ribbons were ground into fine powders (<50 µm) and then sintered using the DC hot-pressing equipment at 923 K for 5 min under a pressure of 60 MPa, and named melt-spinning samples.

X-ray diffraction (XRD; PANalytical X’Pert Pro, Westborough, MA, USA) analysis with Cu Kα radiation was performed on the melt-spinning ribbons and hot-pressing samples. To study the phase evolution of the spun ribbons upon heating, temperature-dependent XRD was also performed from 323 K to 623 K under argon flow. The volume fraction of each phase was estimated by the reference intensity ratio (RIR) method. The surface microtopography and composition were analyzed using a scanning electronic microscope (SEM; Quanta 200FEG, Thermo Fisher Scientific, Waltham, MA, USA) accompanied by the energy-dispersive X-ray spectroscopy (EDS). The microstructure was also investigated using a transmission electron microscope (TEM; Talos F200x and Tecnai G2 F30, Thermo Fisher Scientific, Waltham, MA, USA).

The total thermal conductivity κ_tot_ was calculated following the relationship κ_tot_ = DdC_p_. The thermal diffusivity coefficient (D) was measured by a laser flash system (Netzsch LFA457, Selb, Germany). The specific heat capacity (C_p_) was measured by a DSC system (Netzsch DSC 404C, Selb, Germany). The density (d) of all samples, determined by the Archimedes method, was higher than 96% theoretical density. The Seebeck coefficient and electrical conductivity were measured on a simultaneous measurement system (ULVAC ZEM-3, Chigasaki, Japan).

To investigate the effects of microstructure on the mechanical properties of *p*-type La-filled skutterudite alloys, we conducted the three-point bending test (Imada GA-10N, Toyohashi, Japan) on both the annealed and melt-spinning samples. Large pellets of 20 mm in diameter and 4 mm in thickness were sintered. Two 18 mm × 3 mm × 3 mm bars from each pellet were cut by the diamond wire saw, and the bars were put on two stainless punches with a distance of 13 mm. The force was applied to the upper punch located in the center of the bar.

## 3. Results and Discussions

### 3.1. The Microstructure of the Annealed Samples

**Figure 1:**
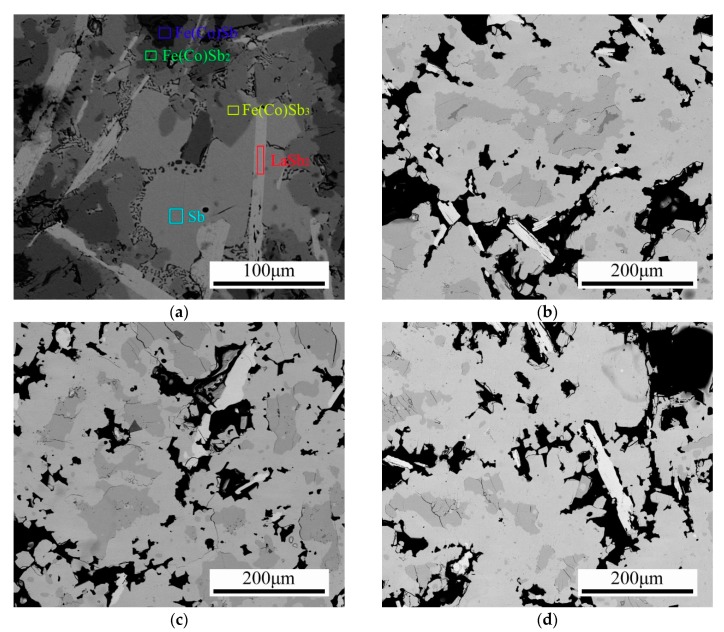

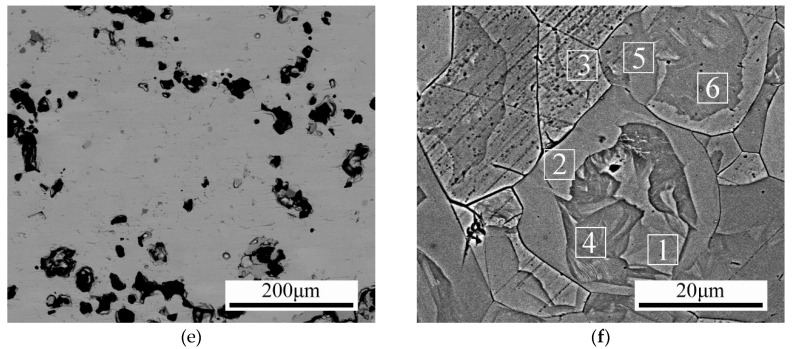
The microstructure of the La_0.8_Ti_0.1_Ga_0.1_Fe_3_CoSb_12_ sample (**a**) as-quenched and annealed at 973 K for (**b**) 10 h, (**c**) 25 h, (**d**) 50 h, (**e**) 100 h and (**f**) the spinodal decomposition zone of the 100 h annealed sample.

Figure 1 shows the microstructure of La_0.8_Ti_0.1_Ga_0.1_Fe_3_CoSb_12_, as-water-quenched and annealed at 973 K for different times. The as-quenched sample (Figure 1a) mainly contained the primary (Fe,Co)Sb dendrites, the first peritectic phase (Fe,Co)Sb_2_, the second peritectic skutterudite phase (Fe,Co)Sb_3_, eutectic Sb and some laminar LaSb_2_ phases. The EDS results indicated that the solid solubility of La in (Fe,Co)Sb and (Fe,Co)Sb_2_ was nearly zero.

As the annealing time increased (Figure 1b–d), the following peritectic transitions took place:

(Fe,Co)Sb + Sb → (Fe,Co)Sb_2_ (1)

(FeCo)Sb_2_ + LaSb_2_ + Sb → La_x_Fe_3_CoSb_12_ (2)

The peritectic skutterudite phase (La_x_Ti_y_Ga_z_Fe_3_CoSb_12_) nucleated on the surface of the intermediate peritectic phase (Fe,Co)Sb_2_, and the growth of the peritectic skutterudite phase was controlled both by the dissolution of LaSb_2_ and the solid-state diffusion of Sb and La. Because of the large size of the primary and intermediate phases, it can be expected that the formation of La_0.8_Ti_0.1_Ga_0.1_Fe_3_CoSb_12_ was time-consuming.

**Figure 2:**
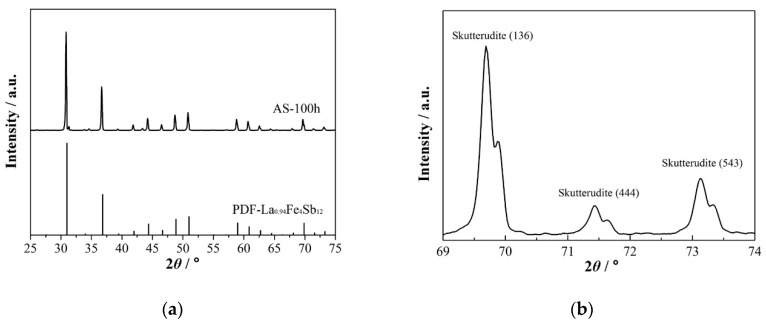
(**a**) The XRD pattern of the sample annealed at 973 K for 100 h. All of the peaks were indexed as the skutterudite phase. (**b**) The enlarged XRD pattern from 2-theta of 69°–74°. The spinodal twin peaks can be clearly seen.

After annealing at 973 K for 100 h, an almost pure skutterudite phase was obtained, as shown in Figure 1e. It is interesting to note that the spinodal decomposition was observed inside each grain, as the bright and dark areas shown in Figure 1f. In contrast to the straight grain boundaries, the phase domains were curved and about 10 μm in size. Figure 2 shows the XRD patterns of the sample annealed at 973 K for 100 h. As shown in Figure 2a, all the peaks were indexed as the skutterudite phase. In the high angle region (as shown in Figure 2b), the appearance of twin peaks was clearly observed, indicating the existence of spinodal decomposition. Table 1 lists the chemical composition of these areas as measured by the EDS methods. According to the EDS result, the bright areas (indicated as 1, 2 and 3) were La- and Fe-rich, while the dark areas (indicated as 4, 5 and 6) were La- and Fe-poor.

### 3.2. The Microstructure of the Melt-Spinning Samples

The thickness of the spun ribbon played a vital role in the formation and evolution of the microstructure inside the spun ribbons. In fact, the cooling rate V_0_ of the spun ribbon can be estimated by [31]:

(3) $$V_{0}=\frac{h}{\rho C_{p}L}(T-T_{A})$$

where *h* is the heat transfer coefficient, *ρ* is the density of the melt, *C*_p_ is the specific heat capacity of the melt, *L* is ribbon thickness, *T* is the temperature of the melt and *T_A_* is the temperature of the copper wheel. Since the thermal parameters of the La-Fe-Co-Sb melt are hard to measure during the melt-spinning process, the high-temperature thermal parameters of La-Fe-Co-Sb are used: *h* ≈ 1 × 10^5^ W/(m^2^·K), *ρ* ≈ 7.7 × 10^3^ kg/m^3^, *C_p_* ≈ 265 J/(kg·K), *T* ≈ 1623 K, and *T_A_* ≈ 298 K.

**Figure 3:**
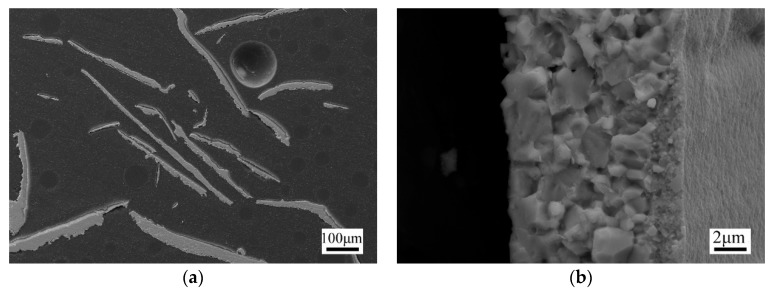

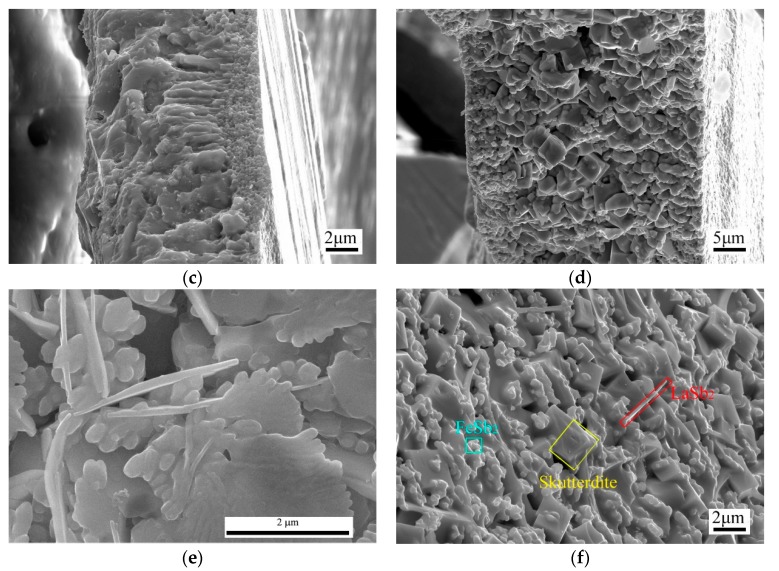
(**a**) The cross-section of various spun ribbons. (**b**) The microstructure of the ribbon with a thickness of 8 µm, nanograins existed near the wheel side. (**c**) The microstructure of the ribbon with a thickness of 13 µm, a columnar crystal layer existed in the center of the ribbon. (**d**) The microstructure of the ribbon with a thickness of 43 µm, the grain size was uniform across the thickness. (**e**) The free side of the 13 µm spun ribbon. The skutterudite dendrites lay parallel to the wheel side. (**f**) The free side of the 43 µm spun ribbon. The volume fraction of LaSb_2_ and (Fe,Co)Sb_2_ phases increased because of the lower cooling rate.

Figure 3a shows the microstructures of the melt spun ribbons with various thicknesses. For the spun ribbon with a thickness of 8 μm, the cooling rate estimated from Equation (3) was about 8.1 × 10^6^ K/s. The microstructure of the wheel side region started with a thin (~1 μm thick) layer of nanograins with a size of 50–100 nm (Figure 3b). Further away from the wheel side, the grains coarsened and achieved sizes up to about 2 μm. For the spun ribbon with a thickness of 13 μm (Figure 3c), the cooling rate estimated from Equation (3) was about 5.0 × 10^6^ K/s. A layer of columnar crystal existed between the nanograin layer and the equiaxed crystal layer. As for the spun ribbon with a thickness of 43 μm (Figure 3d), the cooling rate estimated from Equation (3) was about 1.5 × 10^6^ K/s. The grain size was about 2 μm in the whole sample. 

Some dendrites parallel to the wheel side existed in the free side of the ribbons with a thickness of 13 μm, as shown in Figure 3e. Our EDS results indicate that these dendrites were skutterudite phase. However, for the free side of the spun ribbon with a thickness of 43 μm, the volume fraction of the skutterudite phase decreased while the volume fractions of the (Fe,Co)Sb_2_ and LaSb_2_ intermediate phases increased because of the reduced cooling rate in the thick ribbon. Therefore, the microstructure of the spun ribbons can be well explained by the theory of Kramer et al. [32,33]. Because of the positive thermal gradient across the ribbon thickness, the nucleation rate depends on the undercooling degree and varies through the ribbon thickness, leading to a varying grain size across the ribbon thickness. As the growth front advances, the latent heat released by the formation of the skutterudite phase raises the temperature of the solidified material. Thus, the primary solidified skutterudite phase becomes unstable and tends to undergo the peritectic decomposition. The partial melting of the primary phase results in the dendritic breakup. These dendrites are free to rotate in the liquid because of the surface tension effects. This is why we observed some dendrites on the free side of the ribbons. 

**Figure 4:**
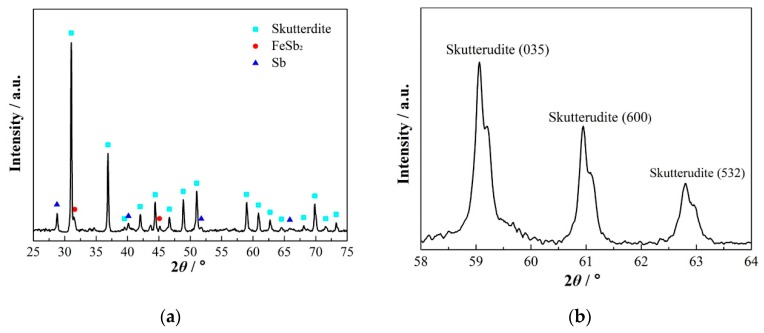
(**a**) The XRD pattern of the spun ribbons, with three phases: the skutterudite phase, the FeSb2 phase and the Sb phase. The main phase is the skutterudite phase. (**b**) The XRD pattern of the spun ribbons in the high 2-theta angle, the spinodal twin peaks of the skutterudite phase, were observed.

The room-temperature XRD pattern (Figure 4a) reveals that most of the spun ribbons are the peritectic skutterudite phase. According to the RIR estimation, the volume fraction of the skutterudite phase in the spun ribbons was about 70%, while it was 25% for the (Fe,Co)Sb_2_ and 5% for Sb phases. This result indicates that the skutterudite phase becomes the primary solidifying phase under the rapid solidification of melt-spinning. Interestingly, spinodal decomposition was also identified in the XRD pattern of the spun ribbons. As shown in Figure 4b, each skutterudite peak possessed a small shoulder on its right side, indicating that there were two skutterudite phases with different lattice constants even in the as-spun ribbons.

**Figure 5:**
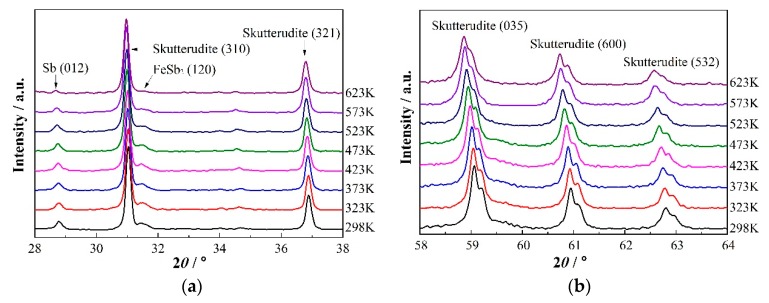

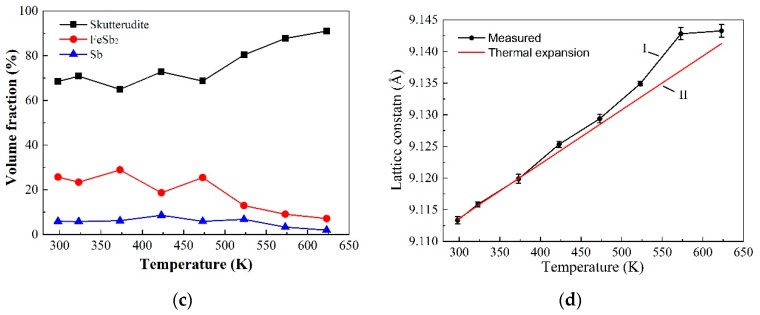
(**a**,**b**) The temperature-dependent XRD patterns of the spun ribbons. (**c**) The volume fraction of various phases at different temperatures. The skutterudite phase started to grow at a temperature as low as 473 K. (**d**) The lattice constant of the La-filled skutterudite phase at different temperatures, starting from 473 K. The increased La filling fraction also lead to the expanded lattice structure.

Figure 5a,b shows the temperature-dependent XRD patterns of the spun ribbons. Upon heating, the volume fraction of the skutterudite phase starts to increase when the temperature is higher than 473 K, as shown in Figure 5c. Such a low peritectic transition temperature of 473 K indicated that the rapid solidification of melt-spinning remarkably reduced the potential barrier of the solid-state phase transitions. As a result, 90% of the spun ribbons transferred to the skutterudite phase at 673 K.

When the temperature was lower than 473K, the spinodal twin peaks shifted to lower angles caused by the thermal expansion. As the temperature increased over 473K, both the filling fraction and the diffusion speed of La in the skutterudite phase increased, leading to an expanded lattice structure (as shown in Figure 5d). According to the above results, we can deduce that the peritectic transition and the spinodal decomposition took place simultaneously in the spun ribbons upon heating. Without any doubt, the synchronism of the peritectic transition and spinodal decomposition resulted in the complex microstructures in the sintered spun ribbons, as described in the following section.

**Figure 6:**
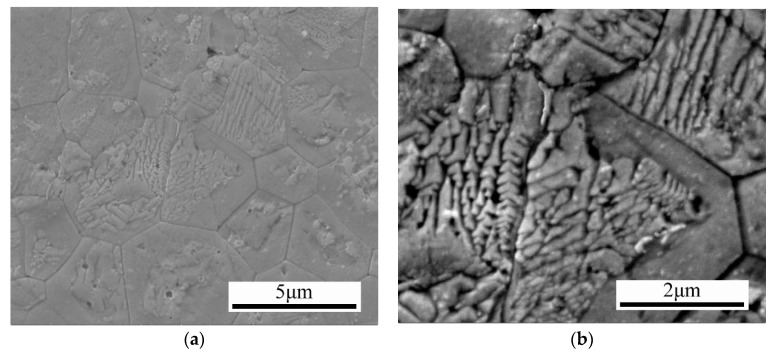

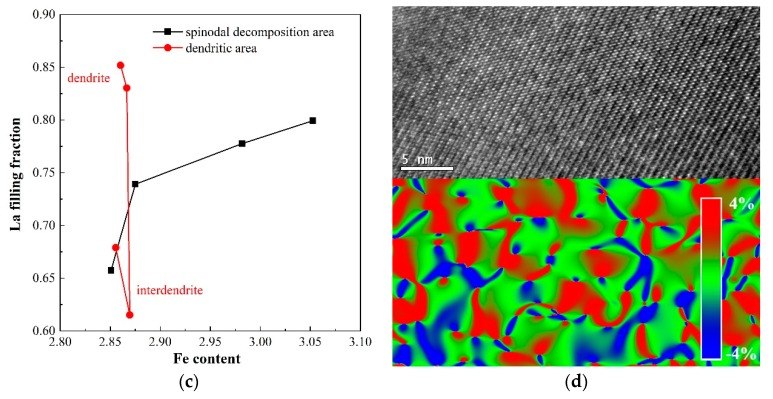
(**a**) The microstructure of the DC hot-pressed samples at low magnification. (**b**) The submicron-scale dendritic segregation and the nanoscale spinodal decomposition zones. (**c**) The La filling fraction in the dendritic segregation and spinodal decomposition zones. (**d**) the high-resolution transmission electron microscopy (HRTEM) image in the grains and the corresponding strain field calculated by the geometrical phase analysis method.

The spun ribbons were pulverized and densified by the DC hot-pressing method. Figure 6a shows the microstructure of the as-sintered samples. High phase purity skutterudites were obtained after the hot-pressing. The grain size was about 3 μm, which was just a little larger than that of the spun ribbons. Interestingly, both dendritic segregation and spinodal decomposition were observed inside the skutterudite grains, as shown in Figure 6b. The spinodal decomposition zones existed in the outermost layer of the grains, while the skutterudite dendrites formed during the melt-spinning process remained in the center of the grains. The dendrite arm space was about 500 nm, while it was about 200 nm for the second dendrite arm space.

Figure 6c shows the EDS result of both the dendritic segregation and spinodal decomposition zones. In the spinodal decomposition zone, the La filling fraction increased linearly with the increasing Fe concentration. On the contrary, in the dendritic segregation zone, only the La filling fraction varied. The La filling fraction in the center of the dendrite arm was higher than that in the inter-arm areas. 

Since the lattice constant of the skutterudite phase increased linearly with the La filling fraction, both the dendritic segregation and the spinodal decomposition generated elastic strain field fluctuation. In fact, Figure 6d shows the HRTEM image and the strain field fluctuation (calculated by the geometrical phase analysis method) of the melt-spinning sample. Such a strain field fluctuation will enhance the scattering of phonons and result in a lower lattice thermal conductivity [27,28,34,35,36]. We also studied the microstructure evolution of CoSb_3_-basde n-type skutterudite alloys fabricated by the current method. Our results indicated that the same ideas do govern the skutterudite microstructure in general. The detailed results will be reported in our future manuscript.

### 3.3. The Thermoelectric Properties

**Figure 7:**
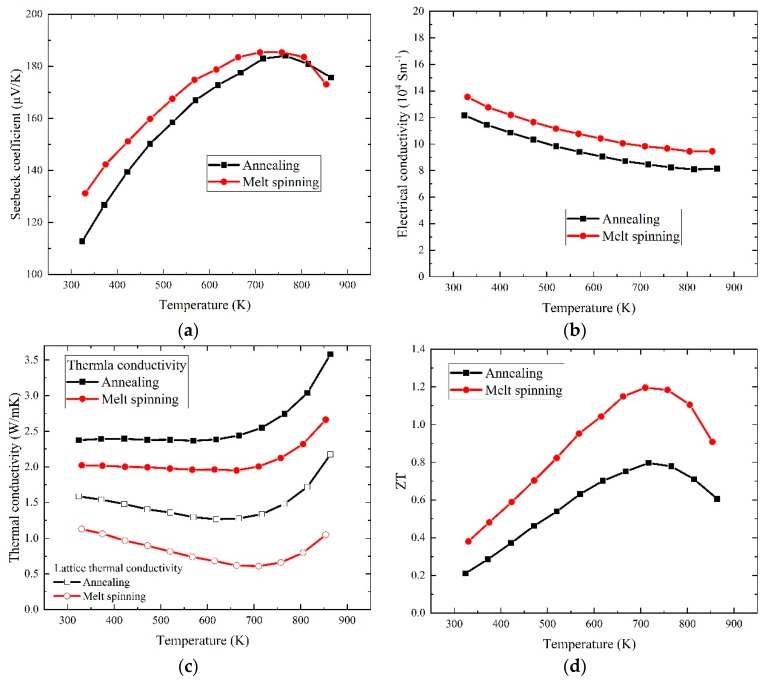
The temperature-dependent thermoelectric properties of the annealed and melt-spinning samples (**a**) the Seebeck coefficient, (**b**) the electrical conductivity, (**c**) the thermal conductivity and (**d**) the dimensionless figure of merit, *ZT*.

Figure 7 shows the temperature dependence of the thermoelectric transport properties of the melt-spinning and annealed samples. The Seebeck coefficient of the melt-spinning sample was very close to that of the annealing samples in the whole measurement temperature range, as shown in Figure 7a. On the other hand, the electrical conductivity of the melt-spinning sample was higher than that of the annealed sample in the whole measurement temperature range, as shown in Figure 7b. The almost identical Seebeck coefficient and higher electrical conductivity resulted in an improved power factor in the melt-spinning sample. It is generally believed that the improved power factor is caused by the aligned valance band maximum (VBM) of Sb-based skutterudites [37,38]. In the melt-spinning samples, the dendritic segregation and spinodal decomposition resulted in the inhomogeneous distribution of the La and Fe/Co cations; this kind of inhomogeneity leads to the conduction band minimum (CBM) fluctuation and keeps the VBM aligned. Thus, the major carriers (holes) were unaffected in the melt-spinning samples, while the minor carriers (electrons) were further scattered by the CBM fluctuation [28]. Therefore, the improved power factor existed in the melt-spinning sample.

The thermal conductivity of the samples is shown in Figure 7c. Compared to the annealed samples, the total thermal conductivity of the melt-spinning sample was lowered remarkably. Using the Wiedemann-Franz law κ_e_ = LσT, the lattice thermal conductivity was estimated by subtracting the electronic thermal conductivity from the total thermal conductivity. Here, the Lorenz constant L was taken as 2 × 10^−8^ WΩ K^−2^ [11]. The result showed that the lattice thermal conductivity of the melt-spinning samples was about 30% lower than that of the annealed samples. The enhanced phonon scattering by the strain field fluctuation was clearly demonstrated.

Figure 7d shows the *ZT* values of the melt-spinning and annealed samples. Because of the increased power factor and reduced lattice thermal conductivity, a maximum *ZT* value of about 1.20 in the melt-spinning sample was obtained, which was about 50% higher than that of the annealed sample.

### 3.4. The Mechanical Properties

**Figure 8:**
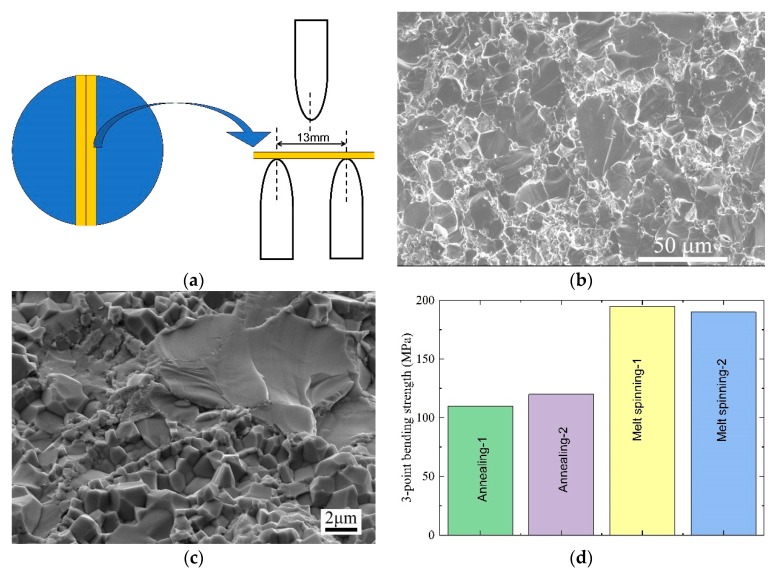
(**a**) The schematic representation of the three-point bending strength measurement set up. (**b**) The fracture microstructure of the annealed sample represented a typical transgranular fracture. (**c**) The fracture microstructure of the melt spinning sample represented a typical intergranular fracture. (**d**) The three-point bending strength. The grain-boundary strengthening greatly improved the three-point bending strength of the melt-spinning samples.

Figure 8a shows the schematic representation of the three-point bending test set up. The three-point bending strength σ*_f_* is calculated as [39]:

(4) $$\sigma_{f}=\frac{3F_{\max}L}{2ab^{2}}$$

where *F*_max_ is the maximum force applied on the upper punch, *a* is the width of the bar, *b* is the height of the bar and *L* is the distance between the two lower punches.

Figure 8b shows the fracture microstructure of the annealed sample. A typical transgranular fracture was clearly observed because of the large grain size (~30 µm). On the contrary, Figure 8c shows that a typical intergranular fracture was found for the melt-spinning sample because of the decreased grain size (~2 µm). A maximum three-point bending strength of about 195MPa in the melt-spinning sample was obtained, which was about 70% higher than that of the annealed sample (Figure 8d). Therefore, the grain boundary strengthening was clearly demonstrated in the melt-spinning sample.

## 4. Conclusions

We have studied the microstructure evolution and mechanical properties of *p*-type La-filled skutterudite alloys fabricated by the long-term annealing and melt spinning, followed by the DC hot-pressing approaches. It was found that the rapid solidification plays a four-fold role: (i) The skutterudite phase nucleates directly from the under-cooled melt and grows into submicron dendrites during the melt-spinning process; (ii) the spun ribbons are highly uniform in chemical compositions and start to form nanoscale La-rich and La-poor skutterudite phases through spinodal decompositions at temperatures as low as 473 K; (iii) the coexistence of the submicron-scale dendrite segregation and nanoscale spinodal decomposition leads to multiscale strain field fluctuation in the hot-pressed sample, which possesses high thermoelectric performance; (iv) because of the micron-scale grain size, the grain boundary strengthening effects lead to a maximum three-point bending strength of the about 195 MPa in the melt-spinning sample, which is 70% higher than that of the annealed sample.

## Figures and Tables

**Table 1:** The actual chemical compositions of the bright and dark areas in the annealing sample.

No.	Actual Chemical Composition
1	La_0.80_Ti_0.1_Ga_0.1_Fe_3.10_Co_0.90_Sb_12_
2	La_0.75_Ti_0.1_Ga_0.1_Fe_2.90_Co_1.10_Sb_12_
3	La_0.75_Ti_0.1_Ga_0.1_Fe_2.90_Co_1.10_Sb_12_
4	La_0.60_Ti_0.1_Ga_0.1_Fe_2.62_Co_1.38_Sb_12_
5	La_0.70_Ti_0.1_Ga_0.1_Fe_2.82_Co_1.18_Sb_12_
6	La_0.65_Ti_0.1_Ga_0.1_Fe_2.70_Co_1.30_Sb_12_
